# Endocarditis-TR: Diagnosis, treatment, and prognosis of the infective endocarditis patients admitting tertiary centres of Turkey

**DOI:** 10.3906/sag-2105-118

**Published:** 2021-12-17

**Authors:** Ali Nazmi ÇALIK, Özlem Arıcan ÖZLÜK, Mehmet Baran KARATAŞ, Yiğit ÇANGA, Semih EREN, Görkem AYHAN, Ayşe AKDENİZ, Ali PALİCE, Aytaç CANDEMİR, Şükrü AKYÜZ, Mehdi ZOGHİ, Ömer KOZAN

**Affiliations:** 1 Department of Cardiology, Faculty of Medicine, Dr. Siyami Ersek Thoracic and Cardiovascular Surgery Training and Research Hospital, University of Health Sciences, İstanbul, Turkey; 2 Department of Cardiology, Faculty of Medicine, Bursa Yüksek İhtisas Training and Research Hospital, University of Health Sciences, Bursa, Turkey; 3 Department of Cardiology, Faculty of Medicine, Ege University, İzmir, Turkey; 4 Department of Cardiology, Faculty of Medicine, Başkent University, İstanbul, Turkey

**Keywords:** Infective endocarditis, in-hospital mortality, heart valve disease

## Abstract

**Background/aim:**

Infective endocarditis (IE) is still a significant cause of morbidity and mortality among cardiovascular diseases. ENDOCARDITIS-TR study aims to evaluate the compliance of the diagnostic and therapeutic methods being used in Turkey with current guidelines.

**Materials and methods:**

The ENDOCARDITIS-TR trial is a multicentre, prospective, observational study consisting of patients admitted to tertiary centres with a definite diagnose of IE. In addition to the demographic, clinical, microbiological, and echocardiographic findings of the patients, adverse events, indications for surgery, and in-hospital mortality were recorded during a 2-year time interval.

**Results:**

A total of 208 IE patients from 7 tertiary centres in Turkey were enrolled in the study. The study population included 125 (60.1%) native valve IE (NVE), 65 (31.3%) prosthetic IE (PVIE), and 18 (8.7%) intracardiac device-related IE (CDRIE). One hundred thirty-five patients (64.9%) were culture positive, and the most frequent pathogenic agent was methicillin-susceptible Staphylococcus aureus (MSSA) (18.3%). Among 155 (74.5%) patients with an indication for surgery, only 87 (56.1%) patients underwent surgery. The all-cause mortality rate was 29.3% in-hospital follow-up. Multivariable Cox regression analysis revealed that absence of surgery when indicated (HR: 3.29 95% CI: 0.93–11.64 p = 0.05), albumin level at admission (HR: 0.46 95% CI: 0.29–0.73 P < 0.01), abscess formation (HR: 2.11 95% CI: 1.01–4.38 p = 0.04) and systemic embolism (HR: 1.78 95% CI: 1.05–3.02 p = 0.03) were ascertained independent predictors of in-hospital all-cause mortality.

**Conclusion:**

The short-term results of the ENDOCARDITIS-TR trial showed the high frequency of staphylococcal IE, relatively high in-hospital mortality rates, shortage of surgical treatment despite guideline-based surgical indications and low usage of novel imaging techniques. The results of this study will provide a better insight to physicians in respect to their adherence to clinical practice guidelines.

## 1. Introduction

Despite advances in diagnostic and therapeutic approaches, infective endocarditis (IE) remains a significant cause of morbidity and mortality among cardiovascular diseases [1]. The disease’s poor outcome necessitates not only to recognize the clinical picture and initiate appropriate antibiotic therapy immediately but also to define the prognostic predictors better.

For that purpose, novel strategies such as additional noninvasive imaging modalities, including multislice computed tomography (MSCT), magnetic resonance imaging, nuclear imaging, and therapeutic strategies consisting of early surgery of complicated cases, have been recommended by the European Society of Cardiology (ESC) guidelines [2]. Nonetheless, it is uncertain whether these recommendations are being implemented in our daily practice in Turkey.

ENDOCARDITIS-TR study aims to evaluate the compliance of the diagnostic and therapeutic methods being used in Turkey with current guidelines. Also, epidemiological, clinical, microbiological, and prognostic features of the IE patients have been assessed.

## 2. Materials and methods

### 2.1. Study design and population

The ENDOCARDITIS-TR trial is a multi-centre, prospective, observational study consisting of patients referred to or admitted to tertiary centres and diagnosed with definite IE following ESC 2015 IE diagnostic criteria. Between 1 February 2018 and 1 February 2020, predefined centres recruited patients presenting with IE and older than 18 years old in the study. Informed consent was obtained from all participants.

### 2.2. Data collection and clinical follow-up

In addition to the patients’ demographic, clinical and microbiological findings, adverse events, indications for surgery, and in-hospital mortality were recorded during a 2-year time interval.

### 2.3. Echocardiography and other imaging tools

The data regarding imaging techniques such as transthoracic and transesophageal echocardiography, multislice computed tomography (MSCT), and nuclear imaging findings were noted.

### 2.4. Definitions and microbiological parameters

Following the 2015 ESC guidelines for the management of infective endocarditis, only the patients having ‘definitive IE diagnosis’ were included in our study. For this reason, at least three sets were taken at 30-min intervals, each containing 10 mL of blood, and were incubated in both aerobic and anaerobic atmospheres. Blood sampling was obtained from a peripheral vein instead of a central venous catheter (because of the risk of contamination) using a meticulous sterile technique.

Transient ischemic attacks (TIAs) have been defined as brief episodes of neurologic dysfunction resulting from focal cerebral ischemia not associated with permanent cerebral infarction, while ischemic stroke was defined as an episode of neurological dysfunction caused by focal cerebral, spinal, or retinal infarction [3,4].

### 2.5. Statistical analysis

All data were presented as a mean ± SD for variables with normal distribution or a median [inter-quantile range] for variables with nonnormal distribution. Categorical variables were reported as numbers and percentages. Continuous variables were checked for the normal distribution assumption using Kolmogorov-Smirnov statistics. Categorical variables were tested by Pearson’s χ2 test and Fisher’s Exact Test. Differences between groups were evaluated using the Mann–Whitney U test or the Student t-test, when appropriate. The comparison of three groups, where indicated, was performed by using one-way ANOVA or Kruskal Wallis tests following their distribution. A further Tukey posthoc analysis was performed for the variables that were considered statistically significant. Univariable and multivariable Cox regression analyses were performed to investigate the independent correlates of all-cause mortality. As a result of the univariable Cox regression analyses, variables that have P values < 0.10 were included in the multivariable Cox regression analyses. P-values were two-sided, and values <0.05 were considered statistically significant. Since the main objective of our study was to evaluate the association between in-hospital mortality and the situation of not receiving surgical intervention even there is a guideline-based indication, the power of the study was assessed using this parameter. The power of the study and the minimum number of patients to be included were calculated following the interim analysis results consisting of the assessment of the first 50 patients enrolled. Considering the type 1 error rate of 0.05 and to achieve 80% power, we calculated that a minimum of 98 patients would be required to claim significant results. All statistical studies were carried out using Statistical Package for Social Sciences software (SPSS 22.0 for Windows, SPSS Inc., Chicago, Illinois)

The study was approved by the local ethics committee (HNEAH-KAEK 2017/KK/152), and it was performed according to the principles of the Declaration of Helsinki.

## 3. Results

A total of 208 infective endocarditis (IE) patients from 7 tertiary centres located in different Turkey regions were enrolled in the study. Demographic and clinical characteristics of patients were summarized in Table 1. The study population included 125 (60.1%) native valve IE (NVE), 65 (31.3%) prosthetic IE (PVIE), and 18 (8.7%) intracardiac device-related IE (CDRIE). The mean age was 58.3 ± 14.8 years and was similar between the three groups. Eighty-three patients (39.9%) were female, and the frequency of the female gender was high in the PVIE group (p = 0.01). While the frequency of coronary artery disease (CAD) and heart failure (HF) was higher in the CDRIE group, atrial fibrillation (AF) was higher in the PVIE group. The groups were comparable in terms of other demographic features, clinical presentation, and symptoms (Table 2). The most frequent symptom on admission was fever in the study population (72.6%).

One hundred thirty-five patients (64.9%) were culture positive, and the most frequent pathogenic agent was methicillin-susceptible Staphylococcus aureus (MSSA) in the overall study group (18.3%). While the frequency of the MSSA and methicillin-resistant Staphylococcus aureus (MRSA) were numerically higher in the CDRIE group, streptococcus viridans were numerically higher in the NVE group. Candida IE was observed only in the NVE group (6 patients) (Table 3). TEE was performed in 189 patients (91%). Fluorodeoxyglucose-positron emission tomography (FDG PET/CT) usage as an imaging modality was relatively low in our study population (5%). (Table 4)

Among 155 (74.5%) patients with an indication for surgery according to the ESC guidelines, only 87 (56.1%) patients underwent surgery. Surgery was not performed in 68 (43.9%) patients despite an indication. Particularly in the PVIE group, cardiac surgery was only performed in 12 out of 40 (30%) patients who indicated surgical treatment. Regarding the CDRIE group, lead extraction, either percutaneously or surgically, was successfully performed in 13 out of 14 patients. The most common indication for cardiac surgery was HF (57.2%), and it was significantly higher in the NVE group compared to other types of IE (p < 0.01). (Table 5)

The all-cause mortality rate was 29.3% in-hospital follow-up. The mean in-hospital follow-up time was 37 days, including four day-stay in the intensive care unit (ICU), with no differences between the groups. (Table 6)

We performed univariable and multivariable binary regression analysis for all variables to identify the independent predictors of all-cause mortality. In univariable regression analyses, age, abscess formation, albumin level at admission, heart failure, uncontrolled infection, systemic embolism, and presence or absence of cardiac surgery when indicated were found to be correlated with all-cause mortality. (Table 7)

After applying these variables into the multivariable Cox regression analysis, absence of surgery when indicated (HR: 3.29 95% CI: 0.93–11.64 p = 0.05), albumin level at admission (HR: 0.46 95% CI: 0.29–0.73 p < 0.01), abscess formation (HR: 2.11 95% CI: 1.01–4.38 p = 0.04) and systemic embolism (HR: 1.78 95% CI: 1.05–3.02 p = 0.03) were ascertained as independent predictors of in-hospital all-cause mortality (Table 7).

Table 8 indicates the comparison of demographic and clinical features between in-hospital death and survival group. Besides being older than the survivors, the albumin levels were found to be significantly lower in the mortality group. IE complications requiring surgery such as heart failure, uncontrolled infection, systemic embolism and abscess were more frequent in the mortality group.

**Figure 1:**
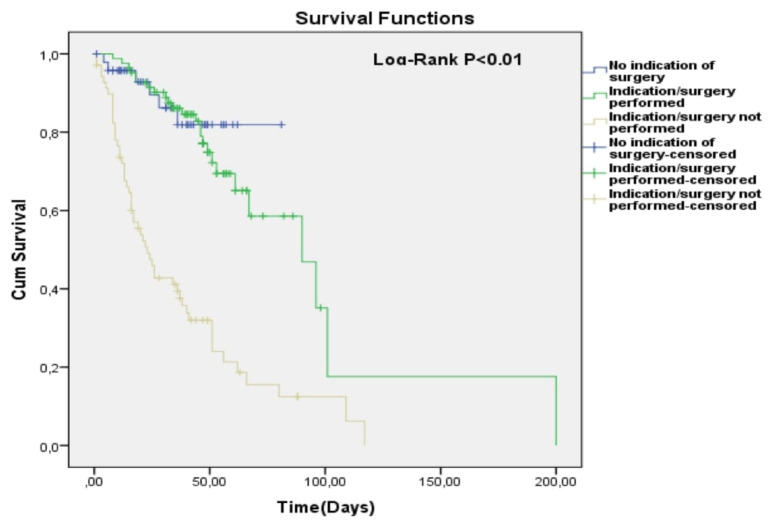
Cumulative survival in three subgroups according to the presence or absence of an indication of surgery and whether surgery was performed or not.

**Figure 2:**
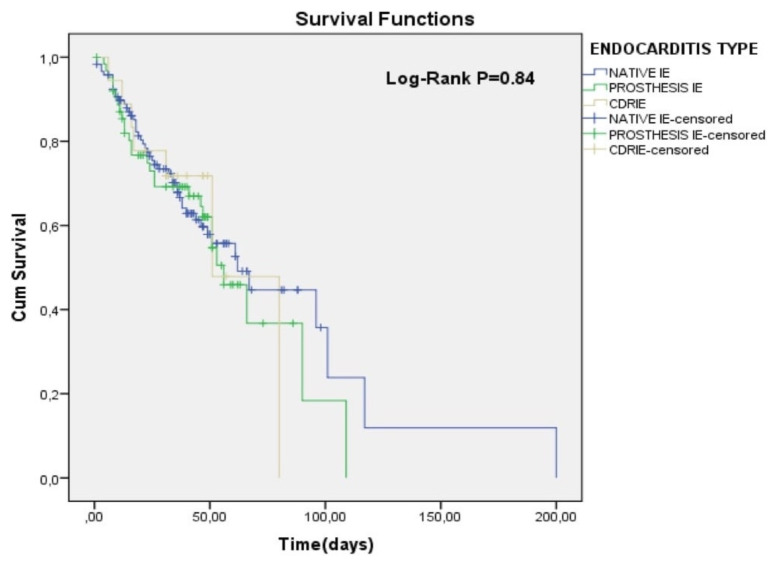
The Kaplan-Meier curves for all-cause in-hospital mortality according to the type of endocarditis.

In Kaplan-Meier curves, patients who did not undergo surgery despite an indication had a significantly higher risk for total mortality compared to other patients having no indication for surgery or undergoing surgery when there is an indication (Log-Rank p < 0.01) (Figure 1). According to the survival table, Kaplan-Meier estimates of 60-days cumulative survival rates between groups (no indication of surgery, indication/surgery performed, indication/surgery not performed) were 87%, 65%, and 21%, respectively. Also, according to the Kaplan-Meier survival analysis, total mortality risk did not differ between groups in terms of endocarditis type (Log-Rank p = 0.84) (Figure 2).

## 4. Discussion

Our study’s most remarkable findings are: (i) The in-hospital mortality rate was extremely high, with 29.3% (ii) Surgery was not performed in 43.9% of the patients despite an indication. Even in the PVIE group, cardiac surgery was performed only in 30 % of the patients who indicated surgical treatment. (iii) FDG PET/CT was used for only 5% of the patients in our study population. (iv) The absence of surgery when indicated, albumin level at admission, abscess formation, and systemic embolism were the independent predictors of in-hospital all-cause mortality. Although there were some previous reports from our country about IE [5–7], this study aimed to investigate the changing patterns of IE’s management, epidemiology, and outcomes.

First, our cohort’s mean age was 58.3 years, with comparable ages reported in the EURO-ENDO registry in 2019 [8] and considerably higher than a previous study (47 years) from Turkey, which was published in 2014 [9]. The previous studies have shown that the mean age of IE patients in developing countries is lower than in high-income countries [10,11]. According to several previous reports, we also know that the median ages of IE patients are progressively increased over the years [8,12–14]. The main underlying cause of the increasing median age of IE patients according to the country’s development level is the difference in risk factor profile. For instance, rheumatic heart disease is a prominent risk factor for infective endocarditis in young people living in developing countries. In developed countries, people’s life expectancy is high, so indwelling cardiac devices and intravenous lines are widely used, which lead to health-care acquired infective endocarditis as an increasing precursor in this aged population. The mean age of patients with IE in our study is the highest compared to other studies published from Turkey to date. This finding can be explained by the medical advances and the increased reach of health care services by a broader population in the last years.

In our study, the proportion of the IE patients according to the nidus (60.1% NVE, 31.3% PVIE, and 8.7% CDRIE) was also following the EURO-ENDO registry. IE has been increasingly encountered in patients with prosthetic valves and intra-cardiac devices over the past two decades [1,15]. The most frequently identified microorganism group was staphylococci in 38.5% of our culture-positive IE patients. The most frequently observed subspecies in the staphylococci group were MSSA, also compatible with recent studies [1,8,9,15]. Staphylococci, a major cause of healthcare-associated IE, has eclipsed streptococci as the most common cause in many high-income countries. Streptococcal infective endocarditis caused by the oral viridans group is the primary cause of NVE and still the most identified pathogen in low-income countries [16]. Streptococci was the causative agent in only 9.1% of our IE patients. Our culture positivity rate was 64.9%, comparable to some of the previous studies [14,17], but lower than the others [1,8,12,18–20]. About 10% of patients with IE show no growth from blood cultures in developed countries. A median of 68% (range 50–84%) of IE causes has been identified in studies from Turkey [9]. The most common cause of culture-negative IE is prior antimicrobial therapy. Besides, the fastidious organisms such as Brucella, Legionella, Chlamydia, Coxiella, and fungi may be the underlying cause of culture-negative IE. Several reports have shown that blood culture-negative IE rates are much higher in developing countries [11,21–23].

Transthoracic echocardiography and TEE remain to be the mainstay for the diagnosis of IE. There exist three main areas of use for TEE in patients with IE. Firstly, for a definitive diagnosis to be made, TEE is the confirmatory procedure performed even in patients with uncomplicated native valve endocarditis diagnosed on TTE. Secondly, complications of IE include perforations, abscesses, and fistulae are evaluated with TEE. Thirdly, the follow-up of blood culture-negative patients with high clinical suspicion of native valve IE is also made with TEE. TTE and TEE are widely available and were performed 100% and 91% of our patients, respectively. 18F-FDG PET/CT is mainly used as a novel diagnostic tool with a better sensitivity in PVIE than in NVE and CDRIE, mainly when the diagnosis is uncertain. 18F-FDG PET/CT was performed for 16.6% of the patients in the EURO-ENDO registry. There is limited access to 18F-FDG PET/CT in our country, which was performed for only 5% of study patients.

The management of patients with IE requires an “Endocarditis Team” approach for optimized patient care. The Endocarditis Team generally includes cardiologists, cardiothoracic surgeons, and infectious disease specialists. The Endocarditis Team’s role was described in 2015 ESC Guidelines [2] for the IE management comprehensively. One of the most important Endocarditis Team tasks is the selection of appropriate patients and timing for surgery. The current reports indicate that early surgery significantly reduces short and long-term mortality in patients with a guideline-based surgical indication [24,25]. Surgery was performed in more than one-half of the patients with IE in previous reports [26]. The most frequent indications for the surgery were valvular dysfunction leading to HF, uncontrolled infection, and embolism prevention. The most common indication for cardiac surgery was HF, and it was significantly higher in the NVE group than other types of IE in our study. Mortality was particularly high in EURO-ENDO when surgery was indicated but not performed. Similarly, the absence of surgery despite an indication was found to be an independent predictor of all-cause mortality in our study. There was an indication for surgery in 74.5% of our patients, but nearly half of those (43.9%) did not undergo surgery. Even though the previous studies reported that nearly one-quarter of IE patients with indications for surgery did not undergo surgery during the initial hospitalization [27], the rate of absence of surgery when indicated in our study (43.9%) is relatively high compared to similar studies.

The most common reasons for the lack of surgery were fragility, hemodynamic instability, stroke, sepsis, and death before surgery in previous reports. In our study population, the most frequent reasons for not undergoing surgery even an indication were patient refusal due to high-risk surgery and death or suffering from a neurological complication while waiting for the operation.

The all-cause mortality rate was 29.3% in-hospital follow-up of our IE patients. The in-hospital mortality of IE was estimated at around 20% in previous studies [28,29]. Simsek et al. have reported a very high mortality rate (27.8%) in a recent single-centre study from Turkey [9]. They have concluded that the higher mortality rate could be due to the referral of more complicated cases to their referral centre. Acknowledging this plausible reason for our study conducted in Turkey’s referral centres, we can further speculate that the main reason for our very high mortality rate may be due to the very high rate of the patients who did not undergo surgery despite an indication. Therefore, it is not surprising to find the abscess formation and systemic embolism as the independent predictors of in-hospital mortality for our study. They are well-known indications for surgery in patients with IE.

Last but not least, S. aureus has been found as the most frequently identified microorganism in our study, which was associated with a lower likelihood of surgery [29] and high in-hospital mortality [8] in previous studies. This may be another plausible reason for very high mortality rates in our study population.

## 5. Conclusion

The short-term results of the multicentre, prospective, and observational ENDOCARDITIS-TR trial showed the high frequency of staphylococcal IE, relatively high in-hospital mortality rates, shortage of surgical treatment despite guideline-based surgical indications and low usage of novel imaging techniques. The present study, despite its limitations, sheds light on the clinical approach of IE patients in Turkey’s referral centres by evaluating their diagnostic and therapeutic features and clinical outcomes. The results of this study will provide a better insight to physicians in respect to their adherence to clinical practice guidelines.

## 6. Limitations

The main limitations of our study may be listed as follows. Firstly, the study consisted only of high-risk patients admitted or referred to tertiary centres. This may have prevented the study from reflecting population-based outcomes and resulted in a higher rate of in-hospital mortality than expected. Secondly, the data regarding the reasons for the absence of surgical treatment should be better provided. However, this does not change the fact that such a conservative approach was an independent predictor of in-hospital mortality. Thirdly, events occurring during the long-term follow will be evaluated in the future trials of ENDOCARDITIS-TR.

## Figures and Tables

**Table 1:** Patient demographics and clinical characteristics.

	Total	Native	Prosthetic	CDRIE	P

Total, N (%)	208	125 (60.1)	65 (31.3)	18 (8.7)	

Mean age ± SD	58.3 ± 14.8	57.8 ± 14.9	59.3 ± 13.9	58.7 ± 17.1	0.81

Females, N (%)	83 (39.9)	46 (36.8)	34 (52.3)	3 (16.7)	**0.01**

**History of CV diseases, n (%)**					

Coronary artery disease	81 (38.9)	41 (32.8)	29 (44.6)	**11 (61.1)**	**0.04**

Heart failure	48 (23.1)	19 (15.2)	16 (24.6)	13 (72.2)	<0.01

Atrial fibrillation	50 (24.0)	16 (12.8)	31 (47.7)	4 (22.2)	<0.01

Congenital heart disease	15 (7.2)	11 (8.8)	3 (4.6)	1 (5.6)	0.54

Previous endocarditis	17 (8.2)	7 (5.6)	9 (13.8)	1 (5.6)	0.13

Pacemaker	9 (4.3)	-	4 (6.2)	5 (27.8)	<0.01

ICD	6 (2.9)	1 (0.8)	-	5 (27.8)	<0.01

CRT-D	7 (3.4)	-	-	7 (38.9)	-

**Risk Factors, n (%)**					

Hypertension	94 (45.2)	56 (44.8)	30 (46.2)	8 (44.4)	0.98

Chronic renal failure	65 (31.3)	42 (33.6)	17 (26.2)	6 (33.3)	0.56
Hemodialysis	30 (14.4)	23 (18.3)	7 (10.8)	-	0.07

Asthma/COPD	16 (7.7)	8 (6.4)	5 (7.7)	3 (16.7)	0.31

Previous stroke/TIA	42 (20.2)	26 (20.7)	14 (21.5)	3 (16.7	0.89

Malignancy	31 (14.9)	20 (16)	10 (15.4)	1 (5.6)	0.50

Immunosuppressive treatment	19 (9.1)	15 (12)	3 (4.6)	1 (5.6)	0.21

Chronic autoimmune disease	22 (10.6)	14 (11.2)	7 (10.8)	1 (5.6)	0.76

Intravenous drug abuse	21 (10.1)	16 (12.8)	3 (4.6)	2 (11.1)	0.20

Alcohol abuse	21 (10.1)	13 (10.4)	7 (10.8)	1 (5.6)	0.79

Smoking	96 (46.2)	64 (51.2)	25 (38.5)	8 (44.4)	0.29

CDRIE, Cardiac device-related infective endocarditis; ICD, Intracardiac defibrilator;

CRT-D, Cardiac resynchronization therapy with ICD; COPD, Chronic obstructive pulmonary disease;

TIA, Transient ischaemic attack.

**Table 2:** Clinical presentation.

	Total (N = 208)	Native (N = 125)	Prosthetic (N = 65)	CDRIE (N = 18)	P
Fever	151 (72.6)	85 (68)	52 (80)	14 (77.8)	0.18
Constitutional symptoms	143 (68.8)	85 (68)	47 (72.3)	12 (66.7)	0.75
Dyspnea	132 (63.6)	87 (69.6)	36 (55.4)	10 (55.6)	0.14
Chest pain	43 (20.7)	29 (23.2)	10 (15.4)	5 (27.8)	0.38
Stroke-TIA	33 (15.9)	21 (16.8)	10 (15.4)	3 (16.7)	0.98
Syncope	12 (5.8)	7 (5.6)	4 (6.2)	1 (5.6)	0.98
NYHA III–IV	81 (38.9)	48 (38.4)	24 (36.9)	9 (50)	0.62

CDRIE, Cardiac device-related infective endocarditis; TIA, Transient ischaemic attack;

NYHA, New York Heart Association.

**Table 3:** Laboratory parameters and blood cultures.

	Total (N = 208)	Native (N = 125)	Prosthetic (N = 65)	CDRIE (N = 18)	P
***Laboratory parameters***					
C-reactive Protein, mg/dL	8.9 [11]	7.5 [8.6]	7.7 [11.8]	7.8 [14.1]	0.78
Procalsitonin, ng/mL	0.45 [6.4]	0.8 [6.3]	0.3 [21.8]	0.1 [2.6]	0.94
hs-Troponin I, ng/mL	0.03 [0.43]	0.1 [0.5]	0.1 [0.1]	0.04 [1.1]	0.21
Pro-BNP, pg/mL	417 [1074]	459 [1207]	220 [740.2]	640 [541.5]	0.65
WBC, 10^3^/uL	10.9 ± 5.3	11.1 ± 5.3	10.9 ± 5.5	9.6 ± 4.8	0.57
Platelet, 10^3^/uL	235.2 ± 99.2	219.2 ± 77	245.3 ± 95.6	232.4 ± 103.9	0.54
Hemoglobin, g/dL	10.5 ± 2.1	10.4 ± 2.0	10.5 ± 2.1	10.8 ± 2.5	0.75
***Blood Cultures***					
Blood culture (−) patients, n (%)	73 (35.1)	21 (33.8)	25 (38.5)	7 (38.9)	0.54
Blood culture (+) patients, n (%)	135 (64.9)	84 (67.2)	40 (61.5)	11 (61.1)	0.69
MSSA	38 (18.3)	20 (16.0)	14 (21.5)	5 (27.8)	0.31
MRSA	14 (6.7)	9 (7.2)	2 (3.1)	3 (16.7)	0.11
Coagulase (−) stafilococcus	28 (13.5)	18 (14.4)	7 (10.8)	3 (16.7)	0.72
Enterococcus spp.	24 (11.5)	19 (15.2)	6 (9.2)	-	0.15
Streptococcus Viridans	19 (9.1)	14 (11.2)	4 (6.2)	1 (5.6)	0.44
Candida	6 (2.9)	6 (4.8)	-	-	-
Gram-negative bacillus	6 (2.9)	2 (1.6)	4 (6.2)	-	0.39

CDRIE, cardiac device-related infective endocarditis; BNP, Brain natriuretic peptide;

WBC, White blood cells; MSSA, Methicillin-susceptible Staphylococcus aureus;

MRSA, Methicillin-resistant Staphylococcus aureus.

**Table 4:** Imaging methods and findings.

	Total (N = 208)	Native (N = 125)	Prosthetic (N = 65)	CDRIE (N = 18)	P
***Methods, n (%)***					
TTE	208 (100)	125 (100)	65 (100)	18 (100)	-
TOE	189 (90.8)	112 (89.6)	62 (95.4)	15 (83.3)	0.17
FDG PET/CT Scan	10 (4.8)	4 (3.2)	6 (9.2)	-	0.11
***Findings, n (%)***					
≥10 mm vegetation	95 (45.3)	**66 (52.8)**	23 (35.4)	6 (33.3)	**0.04**
Abscess	22 (10.6)	16 (12.8)	6 (9.2)	-	0.23
Pseudoaneurysm	9 (4.3)	2 (1.6)	7 (10.8)		**<0.01**
Paravalvular leakage	23 (11.1)	-	**23 (35.4)**	-	**<0.01**
Prosthetic dehiscence	5 (2.4)	-	**5 (7.7)**	-	**<0.01**

CDRIE, Cardiac device-related infective endocarditis;

TTE, TransThoracic echocardiography; TOE, TransOesophageal echocardiography

**Table 5:** Indications and timing of cardiac surgery.

	Total (N = 208)	Native (N = 125)	Prosthetic (N = 65)	CDRIE (N = 18)	P

**Surgical indication, n (%)**	**155 (74.5)**	**101 (80.8)**	**40 (61.5)**	**14 (77.7)**	**0.02**

Heart failure	119 (57.2)	85 (68)	29 (44.6)	5 (27.8)	**<0.01**

Uncontrolled infection	91 (43.8)	49 (39.2)	30 (46.2)	12 (66.7)	0.08

Systemic embolism	57 (27.4)	37 (29.3)	18 (27.7)	4 (22.2)	0.87

**Cardiac surgery performed**	**87 (56.1)**	**62 (61.3)**	**12 (30)**	**13 (92.8)** *	**<0.01**

<2 week	40	31	1	8	
>2 week	47	31	11	5	

**Cardiac surgery not performed**	**68 (43.9)**	**39 (38.3)**	**28 (70)**	**1 (7.2)** *	**<0.01**

CDRIE, Cardiac device-related infective endocarditis

* 13 out of 14 CDRIE patients underwent successful device removal.

**Table 6:** In-hospital follow-up and causes of mortality.

	Total (N = 208)	Native (N = 125)	Prosthetic (N = 65)	CDRIE (N = 18)	P

**In-hospital follow-up, days**	37.2 ± 25.6	37.5 ± 27.8	36.6 ± 23.3	35.5 ± 18.1	0.49
** ICU follow-up, days**	4 [10]	4 [9]	3.5 [8]	4.5 [10.2]	0.24

**In-hospital mortality, n (%)**	61 (29.3)	38 (30.4)	19 (29.2)	4 (22.2)	0.77

Heart failure	49 (23.5)	34 (27.2)	11 (16.9)	4 (22.2)	0.28

Arrhythmias	23 (11.1)	8 (6.4)	**11 (16.9)**	**4 (22.2)**	**0.02**

Cerebral embolism	32 (15.4)	16 (12.8)	14 (21.5)	2 (11.1)	0.24

Peripheral embolism	13 (6.3)	7 (5.6)	6 (9.2)	-	0.32

Acute myocardial infarction	10 (4.8)	7 (5.6)	2 (3.1)	1 (5.6)	0.73

Sepsis	53 (25.5)	30 (24)	19 (29.2)	4 (22.2)	0.69

Malignancy	6 (2.9)	6 (4.8)	-	-	-

CDRIE, Cardiac device-related infective endocarditis; ICU, Intensive care unit.

**Table 7:** Univariate and multivariate regression analysis for predictors of in-hospital mortality.

		Univariate analysis			Multivariate analysis	
	HR	CI	P	HR	CI	P
Heart failure	4.49	[2.21–9.13]	<0.01	1.01	[0.52–1.94]	0.97
Uncontrolled infection	3.23	[1.73–6.03]	<0.01	1.53	[0.83–2.80]	0.16
Systemic embolism	0.25	[0.13–0.51]	<0.01	**1.78**	**[1.05–3.02]**	**0.03**
Albumin (at admission)	1.03	[1.01–1.06]	<0.01	**0.46**	**[0.29–0.73]**	**<0.01**
Age	2.72	[1.11–6.66]	0.03	1.00	[0.98–1.02]	0.49
Abscess	3.45	[1.81–6.59]	<0.01	**2.11**	**[1.01–4.38]**	**0.04**
Indication for surgery – performed	4.78	[1.09–14.87]	<0.01	0.91	[0.26–3.18]	0.89
Indication for surgery – not performed	3.9	[1.2–12]	<0.01	***3.29***	***[0.93–11.64]***	***0.05***

*P <0.05* was considered as a statistically significant difference, HR, Hazard ratio; CI, Confidence interval.

**Table 8:** Comparison of demographic and clinical features between in-hospital death and survival group.

	In-hospital death (n = 61)	Survival group (n = 147)	P
Age, year	63 ± 13.3	56.4 ± 15	**<0.01**
Male, n (%)	36 (59)	89 (60.5)	0.84
Ischemic heart disease, n (%)	32 (52.5)	74 (50.1)	0.10
History of heart failure, n (%)	17 (27.9)	31 (21.1)	0.29
Atrial fibrillation, n (%)	17 (27.9)	33 (22.4)	0.41
Congenital heart disease, n (%)	3 (4.9)	12 (8.2)	0.41
Previous episode of IE, n (%)	3 (4.9)	14 (9.5)	0.27
Pacemaker, n (%)	3 (4.9)	6 (4.1)	0.79
ICD, n (%)	3 (4.9)	3 (2)	0.26
CRT-D, n (%)	1 (1.6)	6 (4.1)	0.37
Hypertension, n (%)	31 (50.9)	63 (42.9)	0.29
Chronic kidney disease, n (%)	25 (41)	49 (33.3)	0.12
Dialysis, n (%)	12 (19.7)	18 (12.2)	0.16
Asthma/COPD, n (%)	5 (8.2)	11 (7.5)	0.86
Stroke-TIA, n (%)	12 (19.7)	30 (20.4)	0.90
Malignancy, n (%)	5 (8.2)	26 (17.7)	0.08
Connective tissue disorders, n (%)	6 (9.8)	16 (10.9)	0.82
Alcohol abuse, n (%)	7 (11.5)	14 (9.5)	0.67
Smoking, n (%)	26 (42.6)	70 (47.6)	0.51
**Endocarditis Type, n (%)**			
Native valve IE	38 (62.3)	87 (59.2)	0.78
Prosthetic valve IE	19 (31.1)	46 (31.3)	0.56
Cardiac Device-Related IE	4 (6.6)	14 (9.5)	0.49
Surgical Indication, n (%)	59 (96.7)	96 (65.3)	***< 0.01***
Peak Troponin I Value, ng/μL	0.08 [0.9]	0.06 [0.15]	0.15
WBC, th/uL	12.3 ± 6.3	11.8 ± 4.8	0.22
Hemoglobin, g/L	10.4 ± 2.1	10.7 ± 2.1	0.09
Procalcitonin, ng/mL	1.5 [8.1]	1.2 [6.4]	0.06
Serum Creatinine, mg/dL	1.3 [1.6]	1.2 [1.1]	0.05
CRP, mg/dL	11.2 [7.4]	9.2 [11.2]	0.07
Neutrophil, 10^3^/μL	10 ± 4.6	9.8 ± 4.1	0.32
Albumin, g/dL	2.9 ± 0.5	3.3 ± 0.5	***<0.01***
BNP, pg/mL	552.5 [1360]	469 [825.7]	0.06
**Complications of IE, n (%)**			
Heart failure	49 (80.3)	70 (47.6)	***<0.01***
Uncontrolled infection	39 (63.9)	52 (35.4)	***<0.01***
Systemic embolism	28 (45.9)	29 (19.7)	***<0.01***
Abscess	11 (18)	11 (7.5)	***0.02***

IE, Infective Endocarditis; ICD, Intracardiac defibrilator;

CRT-D, Cardiac resynchronization therapy with ICD; COPD, Chronic obstructive pulmonary disease;

TIA, Transient ischaemic attack; WBC, White blood cells;

CRP, C-reactive protein; BNP, Brain natriuretic peptide
